# Alpha-2 agonists for refractory neurological symptoms in pediatric palliative care: a scoping review

**DOI:** 10.3389/fped.2025.1542482

**Published:** 2025-05-14

**Authors:** Annalisa Salerno, Fernando Baratiri, Chiara La Piana, Angelica Bincoletto, Franca Benini, Anna Zanin

**Affiliations:** ^1^ Palliative Care and Pain Service, Department of Women’s and Children’s Health, University of Padua, Padua, Italy; ^2^ Department of Women’s and Children’s Health, University of Padua, Padua, Italy

**Keywords:** alpha-2-agonist agents, refractory neurological symptoms, irritability, dystonia, pediatric palliative care, clonidine, dexmedetomidine, children

## Abstract

**Background:**

Children receiving palliative care often suffer from refractory neurological symptoms. In recent years, there has been a growing interest in the use of alpha-2 agonists as a second- or third-line therapy for severe dystonia and irritability.

**Objectives:**

The aim of this review was to provide an overview of the scientific literature on the use of alpha-2 agonists for the treatment of refractory neurological symptoms in pediatric palliative care, evaluating the evidence available and identifying gaps related to their reported efficacy and safety.

**Methods:**

A scoping review was performed according to the PRISMA extension. A systematic search was conducted in PubMed, Medline, EMBASE, Web of Science, CINAHL, and The Cochrane Library, using terms referring to alpha-2 agonists and neurological symptoms in pediatric palliative care.

**Results:**

Seven articles were identified, including three case reports, two case series, one observational cohort study, and one retrospective analysis. Two drugs (dexmedetomidine, *n* = 4/7, and clonidine, *n* = 3/7) were investigated, encompassing a total of 44 patients aged between 7 months and 18 years. Most patients (95%) initiated treatment in an inpatient setting before transitioning to home care. All patients reported clinical improvement; however, 25% of children treated with clonidine discontinued its use due to ineffectiveness or side effects. No adverse effects were reported with dexmedetomidine use.

**Conclusion:**

Alpha-2 agonists are increasingly being used to manage intractable neurological symptoms in pediatric palliative care. However, evidence regarding their safety profile and effectiveness remains limited, highlighting the need for further research in this area.

## Introduction

Refractory neurological symptoms are frequently observed in children with life-threatening or life-limiting disorders (LLDs) who are eligible for pediatric palliative care (PPC). These children can experience many unpleasant symptoms recently defined as irritability of unknown origin (IUO). This condition includes a wide range of manifestations such as agitation/irritability, insomnia, pain, persistent crying and severe dystonia, that can have a serious impact on the child's and their family's quality of life (1). The etiology of these symptoms is generally multifactorial and, in many cases, remains unclear. Therefore, an accurate diagnosis of potential contributing factors (PCFs) is essential to better understand patient distress and tailor effective therapeutic strategies (2, 3).

Their treatment and management are often demanding and require a multidisciplinary approach (4). When PCFs are unidentified or untreatable, and non-pharmacological interventions (e.g., environmental control, soothing and calming practices, psychological therapies, play/music therapy) have already been implemented without providing relief, pharmacological management guided by PPC specialists becomes mandatory (5, 6).

While common treatment strategies include benzodiazepines, opioids, antipsychotics, gabapentin, antispasmodics, and anticholinergic agents, these medications often fail to provide sufficient symptom control. To date, there is limited evidence on the most effective and efficient medications in refractory situations. Any proposed intervention should be discussed within the broader context of caring for the specific child and their family, considering the impact of treatment on quality of life for both the child and caregiver, as well as factors such as onset, duration, mode of administration, and potential side effects.

A recent brief report (7) on troublesome symptoms in pediatric palliative care identified neuroirritability, dystonia, and sleep disorders as conditions that could most benefit from improved management guidelines.

Alpha-2-adrenergic receptor agonists could be considered for routine usage and “as needed” during exacerbation of these symptoms, especially when dysautonomic manifestations are significant.

*Clonidine* is a partially selective centrally acting adrenergic agonist (α1:α2 ratio 220:1). Initially developed as an antihypertensive agent for adults, it is now used in various pediatric clinical settings (8). Clonidine reduces pain signal transmission by activating presynaptic and postsynaptic α2-adrenoceptors in the dorsal horn, mimicking norepinephrine release from descending inhibitory bulbospinal neurons (9). Being lipid-soluble, it can be administered intravenously or orally, with rapid absorption (onset 30–60 min after ingestion, peak plasma levels at 60–90 min, half-life of 12–33 h, and bioavailability of 75%–90%). Reported side effects include hypotension, bradycardia, dry mouth, and drowsiness (10).

*Dexmedetomidine* is a highly selective α2-adrenoceptor agonist (α2:α1 ratio 1,620:1) with sedative, anxiolytic, sympatholytic, and analgesic-sparing effects. It induces sedation by activating central α2-receptors in the locus coeruleus, resulting in a natural sleep-like state. Patients remain arousable and, due to its peripheral vasoconstrictive and sympatholytic properties, the side effects are mainly hemodynamic (e.g., transient hypertension, hypotension, and bradycardia), while ventilation is unaffected. The most common route of administration is intravenous; however, it is also well absorbed through the intranasal and buccal mucosa. Dexmedetomidine is highly protein-bound in plasma and has a rapid and wide distribution throughout the body. Nonetheless, significant inter-individual variability in its pharmacokinetics has been reported. Compared to clonidine, dexmedetomidine is a more potent sedative due to its greater selectivity for α2-receptors (α2:α1 ratio 1,620:1 vs. 220:1), as the activation of central α1-adrenoceptor counteracts the sedative effects of α2-receptors (11).

In adult palliative care, the use of clonidine and dexmedetomidine is mainly described for sedation, delirium control and as an adjunct in analgesia, especially in severe and intractable cancer-related pain (9, 12–15).

To date, the use of alpha-2 agonist drugs to treat refractory neurological symptoms in children, particularly in palliative care settings, is poorly supported by research. Given the still limited and methodologically variable evidence, we conducted a scoping review to investigate their efficacy and safety for this purpose in PPC settings.

## Materials and methods

### Study design

We performed this scoping review according to the Preferred Reporting Items for Systematic Reviews and Meta-Analyses statement for reporting scoping reviews (PRISMA-ScR) (16).

Scoping reviews are a relatively new, but widely used form of research synthesis with the aim to identify knowledge gaps in a particular field of study and to provide direction to future research priorities (17, 18).

This scoping review's protocol has not been registered or made public.

### Search strategy and selection

We preliminary searched MEDLINE (*via* PubMed), Embase, CINAHL, Scopus and Cochrane for studies on the use of alpha-2-agonist agents such as clonidine and dexmedetomidine on children with intractable neurological symptoms published until March 25, 2024.

In June, we repeated the search including articles published up to 17 June. The research terms included “clonidine”, “dexmedetomidine”, “neuroirritability”, “dystonia”, “crying”, “children”, “newborn”, and “adolescent.” We also reviewed articles from reference lists of studies identified in the literature search. Complete research strategy is more extensively described in Table 1. No filters were applied.

**Table 1:** Research strategy.

Argument	Search Terms
Specified drugs	“Alpha-2 agonists” OR “Adrenergic alpha-2 Receptor Agonists” OR “Adrenergic alpha2 Agonists” OR “Adrenergic alpha-2 Agonists” OR “Agonists Adrenergic alpha-2” OR “ST-155” OR “ST 155” OR Clonidine Gemiton OR Hemiton OR Isoglaucon OR Klofelin OR Clofelin OR Clopheline OR “M-5041T” OR “M 5041T” OR “Catapres” OR Catapresan OR Catapressan OR Dixarit OR Dexmedetomidine OR “MPV-1440” OR “MPV 1440” OR Precedex OR Dexdomitor OR Sedadex OR Sileo OR Cepedex OR Dexdor OR “Dexmedetomidine Hydrochloride” OR “Hydrochloride Dexmedetomidine” OR Igalmi
Symptoms	Neuroirritability OR irritability OR delirium OR agitation OR crying OR insomnia OR dystonia OR dystonicus
Age	Child OR Children OR Newborn* OR Infant OR “Preschool Child” OR “Preschool Children” OR Adolescent* OR Teen* OR Teenager* OR Youth* OR “Adolescent* Female” OR “Female Adolescent*” OR “Adolescent* Male” OR “Male Adolescent*”

### Eligibility criteria

Inclusion and exclusion criteria are listed in Table 2. We included articles that fulfilled the following criteria: (1) patients aged 0–18 years; (2) reporting of specific data on alpha-2-agonist agents for treating refractory neurological symptoms in a context of PPC or in patients potentially eligible for PPC.

**Table 2:** Inclusion and exclusion criteria.

	Inclusion criteria	Exclusion criteria
Population	• Patients aged up to 18 years old • Patients with life-limiting, life-threatening or terminal conditions cared by PPC or eligible for PPC	• Patients over 18 years old • Patients without PPC eligibility criteria
Context	• Pediatric palliative care/hospice • ICU/NICU • Home care	• Operative room
Concept	• Use of alpha-2-agonist agents for treatment of intractable neurological symptoms (agitation, dystonia, insomnia, persistent crying, neuroirritability, IUO)	• Postoperative agitation or delirium and withdrawal syndrome • ICU-related delirium
Study	• Intervention and observational studies, including case reports and case series	• Non-research letters, editorials, seminar reviews, conference abstract, animal studies and non-full text accessible articles

PPC, pediatric palliative Care; ICU, intensive care unit; NICU, neonatal intensive care unit; IUO, irritability of unknown origin.

All studies involving children with a life-limiting, life-threatening disease or terminal condition who were considered eligible for PPC (19) were included, even if they were not explicitly under the care of PPC specialists. Delirium, irritability and dystonia in intensive care unit (ICU) patients were excluded. Post-operative agitation and/or delirium and withdrawal syndrome were also excluded. No restrictions were imposed on language and publication date.

### Study selection and data extraction

Identified studies were exported into Covidence (Veritas Health Innovation, Level 10, 446 Collins St, Melbourne VIC 3000, Australia). Four investigators (A.S., A.B., C.L. and Fe.B.) independently screened first the titles and abstracts and then the full texts of all potentially eligible articles. Each article was screened by at least two investigators for inclusion or exclusion, and disagreements were resolved by a senior assessor (A.Z.).

The data from the selected articles were extracted using a specially designed data extraction table. The information collected included: general data (first author's name, publication date, journal, country, design of study, purpose, sample size and setting), patient data (age, gender, primary disease, comorbidities, prevalent intractable neurological symptom, presence of “do not resuscitate” order) and pharmacological data (alpha-2-agonist agent used, treatment length, posology, route of administration, concurrent treatments, efficacy and adverse events).

Three investigators (A.S., C.L. and F.B.) extracted the data independently to improve consistency. Disagreements were resolved by discussion within the research team and by involving a senior researcher (A. Z.).

### Synthesis method

To summarize the extracted data a table was designed, including information about study designs, settings and populations, interventions/agents used and key findings. After reviewing and discussing the extracted data, we identified several major themes that were deemed worthy of further investigation.

No critical appraisal of the evidence was performed, as it is not considered mandatory for this type of review, the purpose of which is primarily descriptive (20).

## Results

**Figure 1:**
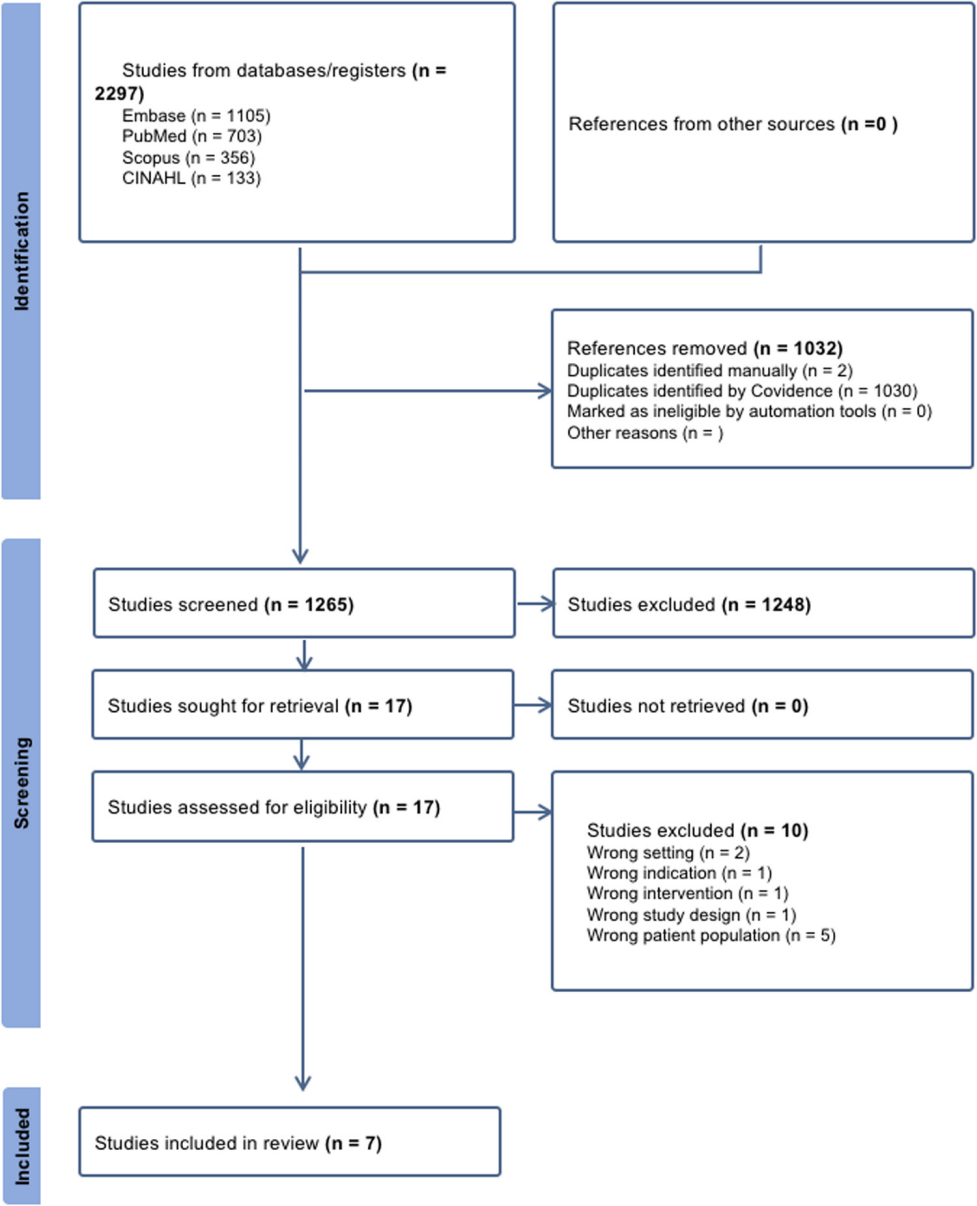
PRISMA flow diagram illustrating the study inclusion process. This diagram is adapted from Page et al. (44). This adaptation is licensed under CC BY 4.0.

Our initial search yielded a total of 2,297 citations. After eliminating duplicates, the remaining 1,265 studies were screened, 17 of which underwent full-text review. A total of 10 papers were excluded during the full-text review for the reasons given in Figure 1. The remaining 7 papers were considered suitable for this scoping review (21–24, 10, 25, 8).

Studies characteristics are summarized in Table 3. The analyzed studies included three case-reports, two case-series, an observational cohort study and a retrospective analysis. No interventional studies were found among the selected papers. All studies were published after 2016: four were conducted in the UK, three in Italy and three in the USA. One case report (23) is also included in one of the case series (20).

**Table 3:** Studies characteristics.

Author/Year/Title	Country	Study design	Aim of the study	Setting and sample (*N*)	Intervention	Key findings (related to study aim and settings)
Bartoletta et al., 2023	USA	Case report	To report the use of dexmedetomidine as a sedative agent for refractory irritability and poor sleep	• Home care • *N* = 1	Administration of IN dexmedetomidine at home	• IN dexmedetomidine appeared safe, reliable and effective • No adverse effects were reported • Limitations: improvement of symptoms reported without objective measurement
“Novel Use of Intranasal Dexmedetomidine for Refractory Irritability in Pediatric Home Care” (21)						
Burns et al., 2017	USA	Observational cohort study	To report the use of dexmedetomidine as a sedative agent for refractory pain and/or agitation in end-of-life care	• Inpatient • *N* = 3	Administration in end of life care of IV dexmedetomidine in PICU and regular units	• IV dexmedetomidine appeared safe and effective • Limitations: small cohort and absence of a control group, improvement of symptoms reported without objective measurement
“The Use of Dexmedetomidine in Pediatric Palliative Care: A Preliminary Study” (22)						
De Zen et al., 2020	Italy	Case report	To report the use of dexmedetomidine as a sedative agent for refractory dystonia	• Inpatient, then home care • *N* = 1	Administration of IN dexmedetomidine, started in hospital and later continued at home	• IN dexmedetomidine appeared safe and effective
“Home Intranasal Dexmedetomidine for Refractory Dystonia in Pediatric Palliative Care” (23)						
De Zen et al., 2023	Italy	Case series	To report the use of dexmedetomidine for children with intractable insomnia, agitation and dystonic states	• Inpatient, then home care • *N* = 9	Administration of dexmedetomidine (IN in 8 patients and IV in 1 patient), started in hospital and later continued at home	• IN and IV dexmedetomidine appeared safe and effective • No adverse events reported • Limitations: small cohort and absence of control group
“Dexmedetomidine at Home for Intractable Dystonia and Insomnia in Children With Special Needs: A Case Series” (24)						
McCluggage et al., 2016	UK	Case report	To report the use of clonidine for refractory dystonia	• Home care • *N* = 1	Administration of continuous SC clonidine, later switched to TD at home	• SC and TD administered clonidine appeared safe and effective • Limitations: improvement of symptoms reported without objective measurement
“Changing from continuous SC to transdermal clonidine to treat dystonia in a teenage boy with end-stage leucodystrophy” (10)						
Nakou et al., 2017	UK	Case series	To report the use of clonidine in the management of severe acute dystonia	• Inpatient • *N* = 5	Administration of enteral and TD clonidine in hospital settings (PICU and regular units)	• High dose clonidine (range 0.1–9 mcg/kg/h) appeared to improve dystonic symptoms (according to DSAP score). • No remarkable impact on respiratory or cardiovascular function
“Safety and efficacy of high-dose enteral, intravenous, and transdermal clonidine for the acute management of severe intractable childhood dystonia and status dystonicus: An illustrative case-series” (25)						
Sayer et al., 2017	UK	Retrospective chart analysis	To determine, as regard to clonidine use in refractory dystonia: • Efficacy in reducing dystonic symptoms • Dose ranges and regimens required • Frequency of side effects	• Outpatient • *N* = 24	Administration of enteral clonidine, started in ambulatory care (Complex Motor Disorders Service) and titrated to the effective dose	• Addition of clonidine to the anti-dystonic regimen improved dystonic symptoms in 85% of included patient • Mild side effects were reported in 50% of patients (drowsiness, insomnia, increased movements)
“Clonidine use in the outpatient management of severe secondary dystonia” (8)						

PPC, pediatric palliative care; IN, intranasal; IV, intravenous; PICU, pediatric intensive care unit; SC, subcutaneous; TD, transdermal; DSAP, dystonia severity action plan.

### Population, setting and symptoms

The age of the population varied significantly across the included studies, ranging from 7 months to 18 years.

Six studies reported patients with congenital or perinatal conditions involving the neurological system, genetic or metabolic disorders (7, 10, 20, 22–24). One study reported mainly oncological and cardiac conditions (21). The presence of “do not resuscitate” (DNR) order was reported by only one study (22), two studies reported some treatment limitations (10, 21) and four did not specify any limitation of care (7, 20, 23, 24).

Different care settings were described in the selected studies. One case report illustrated the intervention completely carried out in a home care setting led by the PPC team (21). Two case reports (10, 23) and a case series (24) described a mixed management, with initiation or dose adjustment in an inpatient/hospital setting and subsequent transition to home care with support from the PPC team.

A retrospective analysis (22) was conducted on patients admitted to PICU and regular inpatient care units managed by the PICU and Pain Medicine teams. Notably, one case series (25) and one observational study (8) were conducted in non-palliative settings, in inpatient and outpatient settings, respectively.

The analyzed studies reported various symptoms that could be defined as refractory neurological symptoms. Refractory dystonia was the most reported sign, accounting for a total of 5 studies; it was described in two case reports, in two case series and in an observational cohort study (8, 10, 23–25). IOU was the main symptom to be controlled in a retrospective analysis involving a cohort of end-of-life patients (22) and in one case report (21) describing a 14-year-old patient with refractory irritability and discomfort. Insomnia was reported as a “challenging symptom” to treat in a case series (24) together with severe dystonia and IOU, but appeared to be almost always reported as an ancillary symptom.

### Alpha-2-agonists for refractory neurological symptoms

Two drugs were investigated in the studies: dexmedetomidine (*n* = 4/7) and clonidine (*n* = 3/7).

Regarding dexmedetomidine, the most common administration routes were intranasal (IN) via a nasal atomizer (*n* = 3/7) (21, 23, 24) and intravenous (IV) infusion (*n* = 2/7) (22, 24). The reported dose for IN administration varied between the studies. A case series (24), which also included a previously published case report by the same authors (23), described a dose ranging from 3–4 mcg/kg/dose for 1–4 times per day (a total of 3–12 mcg/kg/day). Bartoletta et al. (21) described in a case report a starting dose of 0.8 mcg/kg/dose titrated up to 1.5 mcg/kg/dose.

The intravenous use was reported in two studies. A case-series (24) reported a dose of 0.98 mcg/kg/h. An observational cohort study (22) described the suggested regimen to manage the end-of-life symptoms but did not provide specific dosing details for the cohort. The suggested regimen consisted in a bolus dose of 1 mcg/kg administered over 10 min followed by a continuous infusion at 0.1–3 mcg/kg/hour. Treatment duration was reported for IV administration ranging from 1–111 days (22) to 6 months (24) and for IN administration ranging from 1 month–3 years (23). However, no data were reported on the time required to titrate up to an effective dose.

Regarding clonidine, the most common routes of administration described were transdermal (TD) (*n* = 2/7) (10, 25), oral or enteral (*n* = 2/7) (8, 25), followed by IV infusion (*n* = 1/7) (25) and subcutaneous infusion (SC) (*n* = 1/7) (10).

The reported dosage for clonidine differed between studies and routes of administration. Oral clonidine, as reported by Sayer et al. (8) in a retrospective analysis, was administered starting from 1–6 mcg/kg/day (1–2 mcg/kg for 1–3 times per day) up to 75 mcg/kg/day, with an average dosage of 20 mcg/kg/day divided into up to 8 doses. In a case series (25) the use of high-dose clonidine was reported for five neurologically complex patients with status dystonicus, in which clonidine was administered by intravenous infusion in three patients at a maximum dose of 9 mcg/kg/hr. One of them, once stabilized, was switched to clonidine TD, while the other two were switched to enteral clonidine. The two remaining patients were treated directly with enteral clonidine at a maximum dose of 3.9 mcg/kg/h. The authors reported that clonidine was also administered by continuous enteral infusion via feeding tubes. A case-report (10) described the successful use of clonidine SC infusion from an initial dose of 0.59 mcg/kg/h (600 mcg/day) titrated up to 0.74 mcg/kg/h (750 mcg/day) over 7 months, and then switched to TD patches, converting the dose in a 1:1 ratio.

The treatment duration for SC clonidine administration was reported to be over 7 months (10) while TD administration was continued for up to 4 years (25).

Sayer et al. (8) reported a mean time of 9.5 months for clonidine dose optimization to reach the effective dose in an outpatient setting; the other studies did not clearly report these data.

### Concomitant pharmacological treatment

Reported data on the concomitant use of other pharmacological therapies were inconstant. Most studies described patients already taking several therapies [e.g., baclofen, intrathecal baclofen, trihexyphenidyl, benzodiazepines, chloral hydrate, gabapentin (8), tetrabenazine, melatonin, niaprazine, tizanidine, levetiracetam, amitriptyline (24), high dose benzodiazepines, propofol and morphine infusions (22, 25), midazolam and fentanyl patch (10), methadone, morphine, gabapentin, phenobarbital and acetaminophen (21)].

### Efficacy and safety

All of the studies analyzed reported a clinical and/or subjective (reported by parents or caregivers) improvement in symptoms. However, most studies did not use quantitative methods to describe the degree of improvement. Nakou et al. observed an improvement after starting clonidine as measured by the Dystonia Severity Action Plan (DSAP) grade (25). The DSAP helps to measure important clinical variables in dystonic children and to monitor the worsening of condition. Sayer et al. described that clonidine was effective in 83% of patients improving at least one of these 5 areas: seating, sleep, pain, tone and involuntary movements. They also reported that 25% of patients discontinued clonidine due to lack of effect or side effect (8).

No relevant hemodynamic and respiratory adverse effects were reported in the analyzed studies, even for high doses of clonidine administered by different routes (25). Sayer et al. described that nearly half of their study population (13/24) experienced minor adverse effects from clonidine, such as drowsiness (9/24), agitation (3/24) and sleep disturbances (1/24). No adverse events were reported in the dexmedetomidine studies.

## Discussion

The aim of this scoping review was to provide an overview of the available data on alpha-2 agonist medications used to treat refractory neurological symptoms in Pediatric Palliative Care. The seven reviewed papers were all published within the last eight years; however, no interventional studies have been conducted to date. Additionally, no guidelines or standardized protocols were identified.

Unfortunately refractory neurological symptoms are common in pediatric palliative care and represent some of the most challenging symptoms to manage (4), significantly impacting on the quality of life of children and their caregivers (26).

Although there are some reversible causes, their etiology often remains unknown (27), even if a connection with discomfort and pain has been suggested (28).

First line medications such as gabapentin or benzodiazepines are commonly used to treat irritability of unknown origin (IUO), even if evidence is scarce and mostly based on case reports (28, 29). Moreover, several issues related to their use have been reported. For instance, benzodiazepines can often lead to worsened salivation, increased respiratory depression, risk of delirium and paradoxical effects; when they are discontinued after a long period, tolerance and chronic withdrawal symptoms might last for several months (30). Fewer adverse events have been reported for gabapentin, but over-sedation and bradycardia may occasionally occur (31, 32).

As argued in this scoping review, there is a growing awareness among physicians to implement the use of clonidine and dexmedetomidine to manage these burdensome symptoms in pediatric palliative care. Despite limited current evidence, their use seems promising especially when autonomic instability is a prominent feature of irritability (9, 11). According to data from other pediatric settings, both molecules have a good safety profile and may provide less tolerance and fewer pharmacodynamic drug-drug interactions compared to other drugs such as benzodiazepines, opioids and antipsychotics (10, 11). These qualities may be particularly relevant for children who already have a global impairment and who often require polypharmacological drug regimens (33, 34).

Our findings indicated that clonidine and dexmedetomidine were both used successfully, with refractory dystonia as the main indication. However, quantitative methods to assess and describe the improvement were rarely applied. Dexmedetomidine was safely used both intravenously and, more frequently, intranasally (range 1.5–4 mcg/kg/dose) with no reported side effects and the longest follow-up extending to 3 years. The IV dose was reported in only one case (0.98 mcg/kg/h). Clonidine, on the other hand, was also administered intravenously, orally, subcutaneously, and transdermally without severe adverse effects.

As highlighted by Burns et al. and Nakou et al. (23, 25), the use of these molecules, routinely employed in PICU settings for sedation, delirium management, and withdrawal syndromes related to opioids or benzodiazepines, may also be considered as a therapeutic option in cases of unexplained irritability. Another important and encouraging finding from this scoping review is that these drugs can be easily used at home, even for prolonged periods. Regarding clonidine, this finding is also supported by previous experience in the treatment of movement and sleep disorders, especially in children with attention-deficit/hyperactivity disorder (ADHD), developmental delays, autism spectrum disorders and genetic syndromes (35–38).

Furthermore, many routes of administration are available for both agents. The intranasal route offers several advantages: ease and rapidity of administration, non-invasiveness, higher bioavailability without being affected by gastrointestinal dysmotility, reduced pharmacological side effects and shorter time to onset of effects (39, 40). All these elements make clonidine suitable for home management, even as a rescue therapy. Transdermal administration could also be a valid option if the patch is well tolerated. In contrast, subcutaneous and intravenous infusions carry a higher risk of complications and may be more difficult to manage, especially in a home setting (41–43).

However, since children in PPC present a high degree of clinical complexity and numerous comorbidities, we believe that the use of these drugs (which have not been extensively tested yet) should be preceded by a period of observation and parental education by dedicated personnel.

Given the good tolerance and efficacy reported in the limited studies available on this topic, we suggest the possibility of introducing these agents at an earlier stage of the disease trajectory. However, further larger and high-quality studies are warranted to ensure the correct use of these medications by assessing their feasibility, effectiveness, appropriateness, and safety profile.

### Limitations

We found a limited number of studies on alpha-2 agonist medications for treating refractory neurological symptoms in PPC. Most of these studies consisted of case reports or case series with a small number of patients and no randomized controlled trials were found. The efficacy of clonidine and dexmedetomidine was determined on the basis of clinical or subjective improvement, as no quantitative methods or comparisons with control groups were employed.

Dosage and administration routes varied among studies and settings and no standardized protocol was followed.

Regarding safety profiles, the evidence is scarce, and no direct comparison between the two drugs was presented.

## Conclusion

The evidence collected suggests that alpha-2 agonist drugs could represent an effective and promising strategy for the treatment of refractory neurological symptoms in patients with life-threatening and life-limiting diseases. In particular, dexmedetomidine might be more suitable in patients with refractory dystonia while clonidine in movement and sleep disorders. However, due to the paucity of studies, it is not possible to provide a clear recommendation on when to use a specific drug for particular symptoms.

These findings should encourage the design of observational studies with larger sample size and well-designed prospective interventional trials, in order to provide stronger evidence-based recommendations for the application of these drugs. These medications should only be prescribed after careful case-by-case evaluation. Each patient should receive a specific diagnosis, dose and mode of administration tailored to their needs.

Finally, translating and evaluating the use of alpha-2 agonists in other settings, such as pediatric intensive care units, could provide new therapeutic options for managing complex neurological symptoms in critically ill children.
